# Interactions of Calcium Fluctuations during Cardiomyocyte Contraction with Real-Time cAMP Dynamics Detected by FRET

**DOI:** 10.1371/journal.pone.0167974

**Published:** 2016-12-08

**Authors:** Julia U. Sprenger, Nadja I. Bork, Jonas Herting, Thomas H. Fischer, Viacheslav O. Nikolaev

**Affiliations:** 1 Institute of Experimental Cardiovascular Research, University Medical Center Hamburg-Eppendorf, Hamburg, Germany; 2 Clinic of Cardiology and Pulmonology, Heart Research Center Göttingen, University Medical Center Göttingen, Göttingen, Germany; 3 DZHK (German Center for Cardiovascular Research), partner site Hamburg/Kiel/Lübeck, Germany; 4 DZHK (German Center for Cardiovascular Research), partner site Göttingen, Germany; Cinvestav-IPN, MEXICO

## Abstract

Calcium (Ca^2+^) and 3’,5’-cyclic adenosine monophosphate (cAMP) play a critical role for cardiac excitation-contraction-coupling. Both second messengers are known to interact with each other, for example via Ca^2+^-dependent modulation of phosphodiesterase 1 (PDE1) and adenylyl cyclase 5/6 (AC 5/6) activities, which is supposed to occur especially at the local level in distinct subcellular microdomains. Currently, many studies analyze global and local cAMP signaling and its regulation in resting cardiomyocytes devoid of electrical stimulation. For example, Förster resonance energy transfer (FRET) microscopy is a popular approach for visualization of real time cAMP dynamics performed in resting cardiomyocytes to avoid potential contractility-related movement artifacts. However, it is unknown whether such data are comparable with the cell behavior under more physiologically relevant conditions during contraction. Here, we directly compare the cAMP-FRET responses to AC stimulation and PDE inhibition in resting vs. paced adult mouse ventricular cardiomyocytes for both cytosolic and subsarcolemmal microdomains. Interestingly, no significant differences in cAMP dynamics could be detected after β-adrenergic (isoproterenol) stimulation, suggesting low impact of rapidly changing contractile Ca^2+^ concentrations on cytosolic cAMP levels associated with AC activation. However, the contribution of the calcium-dependent PDE1, but not of the Ca^2+^-insensitive PDE4, to the regulation of cAMP levels after forskolin stimulation was significantly increased. This increase could be mimicked by pretreatment of resting cells with Ca^2+^ elevating agents. Ca^2+^ imaging demonstrated significantly higher amplitudes of Ca^2+^ transients in forskolin than in isoproterenol stimulated cells, suggesting that forskolin stimulation might lead to stronger activation of PDE1. In conclusion, changes in intracellular Ca^2+^ during cardiomyocyte contraction dynamically interact with cAMP levels, especially after strong AC stimulation. The use of resting cells for FRET-based measurements of cAMP can be justified under β-adrenergic stimulation, while the reliable analysis of PDE1 effects may require electric field stimulation.

## Introduction

3’,5’-cyclic adenosine monophosphate (cAMP) is a universal second messenger which regulates a plethora of cellular functions [1]. Upon stimulation of G-protein coupled receptors, cAMP production is activated or inhibited via stimulatory and inhibitory G-proteins, respectively. These G-proteins modulate the activity of several families of the cAMP producing enzymes adenylyl cyclases (ACs), which convert adenosine triphosphate to cAMP. The cAMP signals terminate by the action of specific phosphodiesterases (PDEs), enzymes which hydrolyze cAMP to AMP. Real time cAMP dynamics in living cells can be visualized using highly sensitive biosensors based, for example, on Förster resonance energy transfer (FRET). An increasing amount of publications use the FRET approach to investigate cAMP signaling in resting rodent and human cardiomyocytes [2–10].

However, *in vivo*, mammalian heart is under constant contraction, which leads to rapid calcium (Ca^2+^) cycling in all cardiomyocytes during each contraction cycle. Upon depolarization, Ca^2+^ rapidly enters the cell via voltage-gated L-type calcium channels. This in turn triggers Ca^2+^ release from the sarcoplasmic reticulum (SR) via ryanodine receptors, providing a sufficient amount of this ion in the cytosol to activate contractile proteins. In the diastole, there is a Ca^2+^ reuptake back into SR via sarcoplasmic/endoplasmic reticulum calcium ATPase 2 (SERCA2) [11]. Each of these three calcium handling proteins can be positively regulated by the cAMP dependent protein kinase (PKA) which phosphorylates them either directly or via phosphorylation of phospholamban, a small protein which binds to and inhibits SERCA2 function [11,12]. Besides its crucial role in contractility, Ca^2+^ is also known for dynamically regulating intracellular cAMP levels. This occurs either via the stimulation of the Ca^2+^/calmodulin-dependent PDE1 [13] or by inhibition of cardiac AC 5/6 activities [14], all these enzymes are abundantly expressed in the heart [15,16]. Thus, Ca^2+^ and cAMP tightly interact to regulate cardiac excitation contraction coupling (ECC). This interaction is supposed to occur especially in subcellular microdomains formed around calcium handling proteins which might directly impact on contractile function [2,4,17,18]. Furthermore, local subsarcolemmal cAMP levels can affect the activity of L-type calcium channels which is also restricted by a negative feedback loop via PKA mediated phosphorylation of PDE4 [4,19]. To avoid movement artifacts and to increase cell survival during the measurements, the majority of current FRET studies use resting cardiomyocytes to analyze intracellular cAMP dynamics [2–10]. Therefore, it is still unclear whether or not the rapid fluctuations in intracellular Ca^+^ levels during contraction are able to directly affect global and local cAMP levels in the cell. Thus, it remains unknown whether the cAMP imaging data obtained under such conditions truly represent actual cAMP dynamics occurring under more physiological conditions in beating cells.

Here, we sought to answer this question and performed FRET measurements in freshly isolated cardiomyocytes from transgenic Epac1-camps and pmEpac1 cAMP FRET sensor mice [8,20] under electric field stimulation and compared these data to non-paced cells. We could not detect major differences in cAMP signals upon beta-adrenergic receptor (β-AR) stimulation with isoproterenol (ISO), suggesting that rapid fluctuations of Ca^2+^ during contraction are not sufficient to affect overall PDE1 and AC activities. In sharp contrast, the contribution of PDE1 to cAMP degradation after forskolin stimulation was significantly increased in paced cardiomyocytes. This effect could be mimicked in resting cells by preincubation with Ca^2+^ elevating reagents such as thapsigargin and calcium ionophore. Thus, the cAMP dynamics under β-AR stimulation can be reliably studied in resting cells, while the exact analysis of PDE1-associated effects requires paced cardiomyocytes.

## Materials and Methods

### Cardiomyocyte Isolation

All animal work was conducted according to relevant national and international guidelines. Institutional Committee “Tierschutzbüro UMG” and the national authorities LAVES and BGV Hamburg have approved this work. Transgenic mice were custom generated on FVB/NRj background (Janvier Labs, Saint Berthevin, France) as previously described [8,20] and housed (maximum 5 mice per cage) in an open-cage barrier facility under a 12 h light/dark cycle and food/water access ad libitum. A total number of 35 animals was sacrificed for this study. 3–4 month old CAG-Epac1-camps mice ubiquitously expressing the cytosolic cAMP biosensor Epac1-camps under the control of cytomegalovirus enhancer/chicken β-actin promoter [20] or 3–4 month old pmEpac1 mice expressing membrane targeted cAMP sensor in adult cardiomyocytes under the control of α-myosin heavy chain promoter [8] were euthanized by cervical dislocation, and primary adult cardiomyocytes were isolated exactly as previously described [21]. After Ca^2+^ adaptation, cells were plated onto laminin coated coverslips and used for measurements during the next few hours after plating.

### FRET measurements

Coverslips with isolated cardiomyocytes were transferred into a measuring chamber and rinsed once with Tyrode’s solution (4 mM KCl, 149 mM NaCl, 1 mM MgCl_2_, 5 mM HEPES, 10 mM glucose, 1 mM CaCl_2_, pH 7.5). The measuring chamber was then transferred onto a ZEISS AxioObserver A1 epifluorescence microscope equipped with an oil immersion 63x objective, polychrome V light source (TILL Photonics), DV2 DualView (Photometrics) and a CoolSNAP-HQ2 CCD-camera (Visitron Systems). Cells were treated with several cAMP stimulating substances (isoproterenol, rolipram, forskolin and 8-methoxymethyl-3-isobutyl-1-methylxanthine, all from Sigma-Aldrich; or with 3-isobutyl-1-methylxanthin, purchased from AppliChem). Resting cardiomyocytes were pretreated with Ca^2+^ elevating reagents thapsigargin and calcium ionophore A23187 (both from Sigma) for 3 min before starting the FRET measurements to mimic contractility driven increase in Ca^2+^.

Epac1-camps and pmEpac1 expressing cardiomyocytes were excited with 436 nm light. The emission was split into two channels using the DV2 DualView (505dcxr filter) and detected at 535 ± 15 (YFP) and 480 ± 10 (CFP) nm. FRET changes were monitored using VisiView software (Visitron Systems) as the emission ratio of YFP over CFP, calculated as previously described [21] using Origin 8.5 software. For pacing experiments, cardiomyocytes were stimulated at 1 Hz and 20 V with a Myopacer cell stimulator (manufacturer IonOptix). Steady state contraction of cardiomyocytes was determined before and after the experiment and separately confirmed by Ca^2+^ imaging. FRET-based cAMP recordings were usually started 3 min after the beginning of pacing protocol.

### Calcium measurements

Freshly isolated wildtype cardiomyocytes were plated onto laminin coated glass coverslides, rinsed once and then loaded with 1 μM Fura2-AM (Life Technologies) for 15 min at 37°C. After washing the cells thrice with Tyrode solution, measuring chambers were transferred onto a Nikon Eclipse TE2000-U microscope equipped with a fluorescence detection system (IonOptix). Ca^2+^ transients were recorded according to the corresponding FRET experiments. F_340_/F_380_ values in Fura2-AM experiments were calculated from the ratios of 510 nm emission light measured at 340 and 380 nm excitations. Data were analyzed using the IonWizard, GraphPad Prism and Origin 8.5 software.

### Statistical analysis

The values are expressed as means ± SEM. Normal distribution was tested by the Kolmogorov-Smirnov test. Differences between the paced and resting cells or between treated and untreated resting myocytes were analyzed by GraphPad Prism software using one-way ANOVA or Mann-Whitney tests with Bonferroni’s post-hoc test and considered significant at p<0.05. Calcium imaging data were analyzed using one-way ANOVA followed by the Greenhouse-Geisser correction which accounts for the sphericity of the data.

## Results

### β-adrenergic and forskolin stimulated AC effects are not altered in paced vs. resting cardiomyocytes

**Fig 1:**
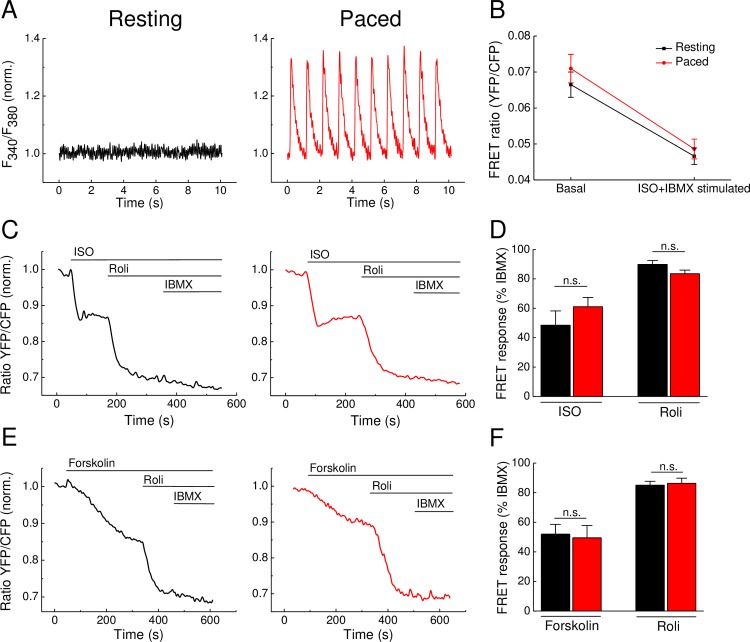
cAMP dynamics in adult mouse ventricular Epac1-camps expressing cardiomyocytes upon treatment with cAMP elevating agents and PDE4 inhibition. **(A)** Representative calcium traces in Fura2-AM loaded Epac1-camps transgenic cardiomyocytes under resting conditions (left) and upon electric field stimulation at 1 Hz (right). **(B)** Non-normalized FRET ratios do not differ between resting and paced Epac1-camps cardiomyocytes under basal and stimulated conditions (isoproterenol—ISO, 100 nM—plus 3-isobutyl-1-methylxathin—IBMX, 100 μM). **(C)** Representative FRET traces from Epac1-camps cardiomyocytes stimulated with the β-AR agonist isoproterenol (ISO, 100 nM) or **(E)** with the direct AC activator forskolin (10 μM) leading to an increase of cAMP visualized as a decrease in the FRET ratio. Inhibition of PDE4 by rolipram (Roli, 10 μM) strongly enhances this effect, whereas the unselective PDE inhibitor IBMX (100 μM) has only little additional effect. **(D and F)** Quantification of the FRET results reveal no significant differences in FRET ratio changes between resting and paced cardiomyocytes. Cells were paced at 1 Hz. Values are means ± SEM; from n = 6 cells isolated from 3 hearts per condition; n.s.—not significant by one-way ANOVA.

To study how cytosolic Ca^2+^ fluctuations associated with cardiomyocyte contraction impact on cAMP dynamics detected by FRET, we measured cAMP responses in resting and paced cardiomyocytes. To ensure that our pacing protocol leads to proper cardiomyocyte contraction and Ca^2+^ transients, we loaded freshly isolated wildtype cardiomyocytes with Fura2-AM. Fig 1A shows that the electrical field stimulation protocol leads to consistent Ca^2+^ transients, while resting cells show no fluctuations in Ca^2+^ signal. Importantly, our pacing protocol altered neither the basal FRET nor the FRET ratio after full stimulation in Epac1-camps expressing cardiomyocytes (Fig 1B), confirming that the FRET sensor properties are not affected by pacing.

Next, we analyzed the effects of contraction on AC activity detected by FRET in Epac1-camps cardiomyocytes. Cells were stimulated either with the β-adrenergic agonist isoproterenol (ISO) to increase the β-AR-dependent AC activity (Fig 1C and 1D) or with the direct AC activator forskolin (Fig 1E and 1F). Both stimulating agents led to an increase in cAMP, visualized by a decrease in the FRET ratio, which was comparable in resting and paced cells. Upon subsequent inhibition of the major cAMP hydrolyzing phosphodiesterase family PDE4 with rolipram, cytosolic cAMP levels increased even further, while the non-selective PDE inhibitor 3-isobutyl-1-methylxanthin (IBMX) added on top of rolipram had only minor additional effect. Interestingly, we could not detect any significant differences between control and paced cardiomyocytes regarding ISO, forskolin and PDE4 inhibitor effects (Fig 1D and 1F).

**Fig 2:**
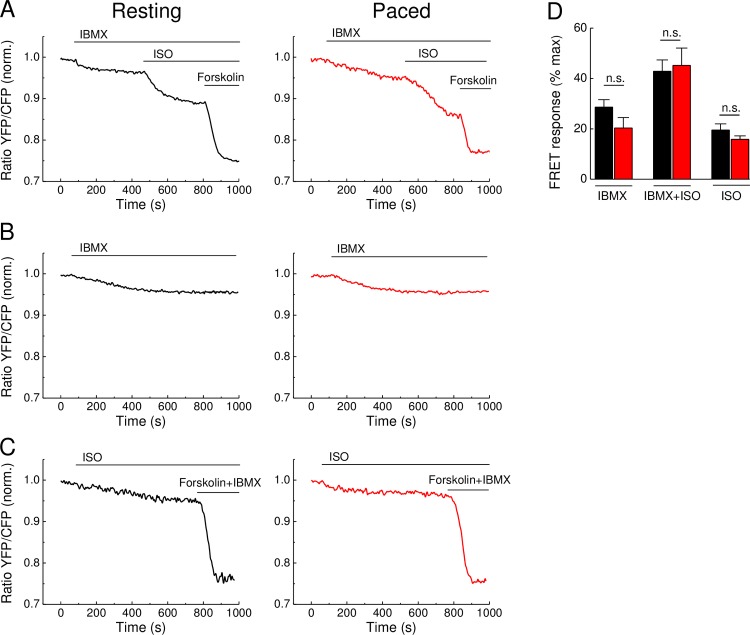
cAMP dynamics in adult mouse cardiomyocytes upon preincubation with IBMX and after low concentration of ISO. **(A)** Representative FRET traces of Epac1-camps cardiomyocytes preincubated with 3-isobutyl-1-methylxanthin (IBMX, 100 μM) and then stimulated with non-saturating concentrations of the β-AR agonist isoproterenol (ISO, 1nM). The AC activator forskolin (10 μM) was used to reach the maximal FRET response. **(B)** Representative control traces (n = 4 and 8 for unpaced and paced, respectively) showing FRET response to IBMX response over the whole time-course involved in these experiments. Representative FRET responses (n = 7 each) to 1 nM ISO applied along (without IBMX prestimulation), followed by forskolin plus IBMX **(D)** Quantification of experiments from A and C shows no significant difference in FRET responses between control and paced cardiomyocytes stimulated with IBMX and ISO. Cells were paced at 1 Hz. Values are means ± SEM, n = 8 cells for each A graph and n = 7 cells for each C graph isolated from 3 hearts per condition; n.s.—not significant by one-way ANOVA.

To ensure that the absence of any measurable effect of pacing was not due to very high degree of AC stimulation by saturating doses of ISO and forskolin or due to variable PDE activities, we analyzed cAMP signals also using non saturating ISO concentrations (1 nM instead of 100 nM) and after PDE inhibition by the unselective PDE inhibitor IBMX. Even in this case, there were no significant differences in the FRET responses when compared in resting and paced Epac1-camps cardiomyocytes (Fig 2). These data suggest that rapid changes in intracellular Ca^2+^ during contraction can significantly affect neither basal nor stimulated AC activities detected by FRET.

### PDE1 contribution to cAMP hydrolysis is affected by intracellular Ca^2+^ fluctuations

**Fig 3:**
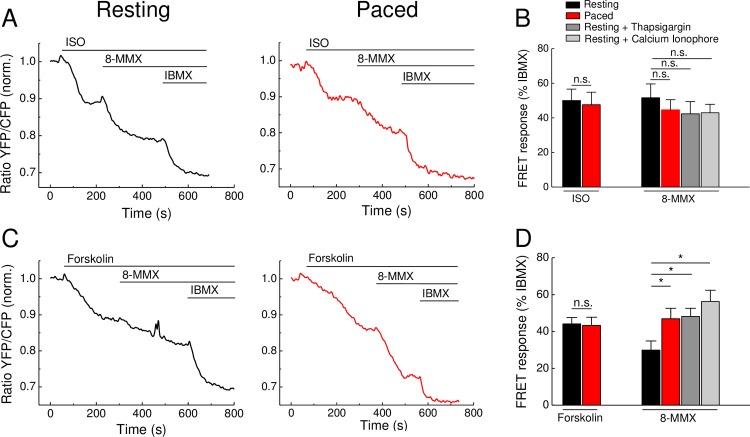
cAMP dynamics in adult mouse cardiomyocytes upon treatment with cAMP elevating agents and PDE1 inhibition. **(A)** Representative FRET traces of Epac1-camps cardiomyocytes stimulated with the β-AR agonist isoproterenol (ISO, 100 nM) or with the direct AC activator forskolin (10 μM) **(C)**. Subsequent application of the PDE1 inhibitor 8-methoxymethyl-3-isobutyl-1-methylxanthine (8-MMX, 30 μM) enhances the cAMP stimulatory effect of ISO and forskolin. Stimulation with the unselective PDE inhibitor 3-isobutyl-1-methylxanthin (IBMX, 100 μM) leads to a further increase of cAMP. **(B and D)** Quantification of experiments shows no significant difference in FRET responses between control and paced cardiomyocytes stimulated with ISO. Forskolin stimulated cardiomyocytes show significant differences in PDE1 contribution to total PDE inhibition which is significantly higher in paced cardiomyocytes as compared to resting cells. Pretreatment of resting cardiomyocytes with calcium elevating reagents such as thapsigargin (100 nM) and calcium ionophore A23187 (10 μM) mimics the effect of field stimulation. Cells were paced at 1 Hz. Values are means ± SEM; n = 6–10 cells isolated from 3 hearts per condition; *—significant difference at p<0.05 by one-way ANOVA; n.s.- not significant.

It is known that PDE1 hydrolytic activity toward cAMP is stimulated by Ca^2+^ via calmodulin [13], so that one can expect a potential decrease of cAMP levels upon Ca^2+^ elevation. Thus, it was important to investigate whether Ca^2+^ fluctuations associated with contraction would have any influence on the contribution of PDE1 into the cAMP signals detected in FRET experiments. Therefore, we performed FRET measurements similar to those shown in Fig 1, but using the PDE1 selective inhibitor 8-methoxymethyl-3-isobutyl-1-methylxanthine (8-MMX) instead of rolipram (Fig 3). In control and paced cardiomyocytes, we detected no significant differences in the amplitude or kinetics of the FRET signals to ISO and 8-MMX applied after ISO, suggesting no effect of rapid Ca^2+^ transients on the overall PDE1 activity after β-AR stimulation (Fig 3A and 3B). Interestingly, prestimulation of cells with the direct AC activator forskolin significantly increased PDE1 contribution to cAMP hydrolysis, as revealed by significantly greater 8-MMX effects in field stimulated compared to resting cardiomyocytes (Fig 3C and 3D). This suggests a rise in PDE1 activity following forskolin stimulation combined with contraction. As expected, this effect could be mimicked by preincubation of resting cardiomyocytes with Ca^2+^ elevating agents such as thapsigargin and calcium ionophore (Fig 3D), suggesting the calcium dependent nature of this response.

### Pacing does not affect subsarcolemmal PDE1 and PDE4 responses after β-adrenergic stimulation

**Fig 4:**
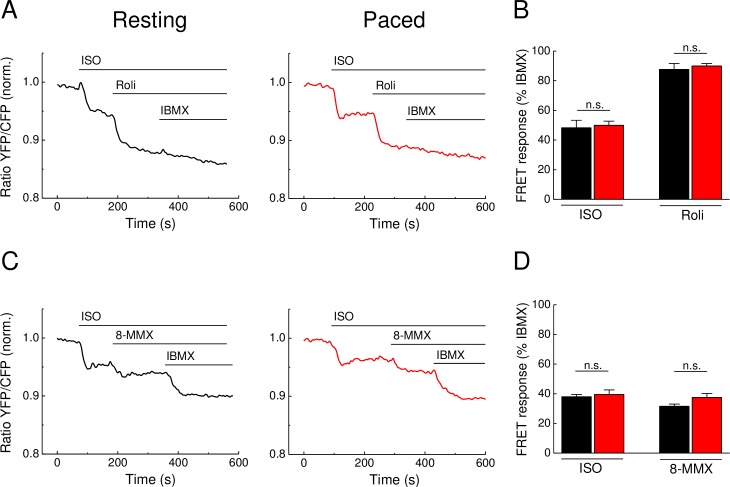
Subsarcolemmal cAMP dynamics in adult mouse ventricular cardiomyocytes transgenically expressing pmEpac1 biosensor. **(A)** Representative FRET traces from pmEpac1 cardiomyocytes stimulated with the β-AR agonist isoproterenol (ISO, 100 nM) and subsequently by the PDE4 inhibitor rolipram (Roli, 10 μM) followed by the unselective PDE inhibitor IBMX (100 μM) as described in Fig 1C and 1D. **(C)** Representative FRET traces from pmEpac1 cardiomyocytes stimulated with ISO (100 nM) and subsequently by the PDE1 inhibitor 8-MMX (30 μM) followed by the unselective PDE inhibitor IBMX (100 μM) as described in Fig 3A and 3B. **(B and D)** Quantification of the FRET results reveal no significant differences in FRET ratio changes between resting and paced cardiomyocytes treated with ISO, rolipram or 8-MMX. Cells were paced at 1 Hz. Values are means ± SEM; from n = 12 and n = 11 cells (unpaced and paced, respectively) isolated from 3 hearts per condition in B and n = 9 cell from 2 hearts each in D; n.s.—not significant by one-way ANOVA.

Since local subsarcolemmal Ca^2+^ and cAMP pools formed in close proximity to calcium handling proteins are even more important for cellular contractility and function, we next used cardiomyocytes expressing the pmEpac1 biosensor, a localized version of Epac1-camps which is specifically targeted to caveolin-rich membrane domains. This sensor is positioned in the T-tubules and caveolae close to L-type calcium channels, a functional microdomain associated with cardiomyocyte contractility [8]. To this end, we sought to test whether Ca^2+^ transients can more prominently affect local subsarcolemmal cAMP dynamics already in the settings of β-adrenergic stimulation combined with PDE4 and PDE1 inhibition. Interestingly, neither ISO and rolipram (Fig 4A and 4B) nor ISO and 8-MMX effects (Fig 4C and 4D) at the membrane were affected by pacing. This suggest that no stronger contraction-associated effect of PDE4 or PDE1 could be revealed in this functionally relevant microdomain.

### Ca^2+^ imaging in stimulated cardiomyocytes reveals a stronger effect of forskolin on Ca^2+^ transient as compared to ISO

**Fig 5:**
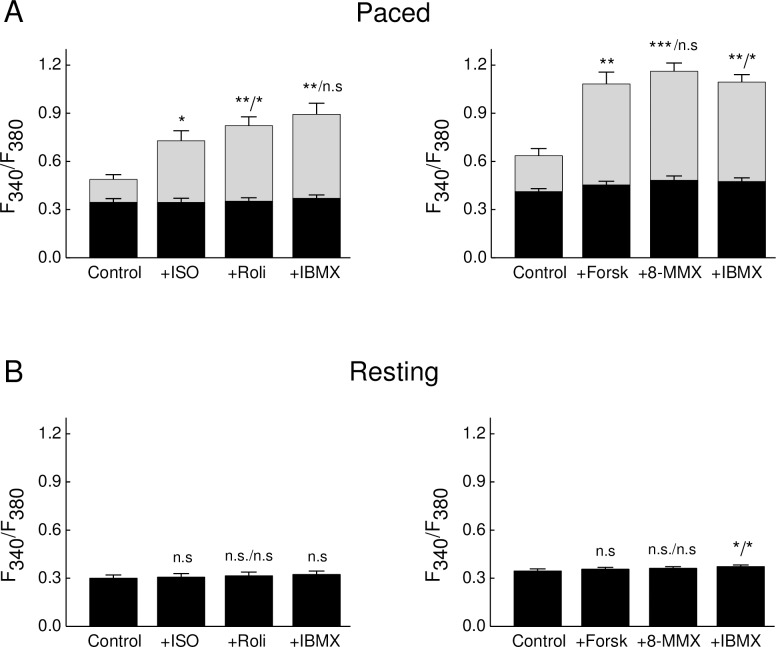
Ca^2+^ transient amplitudes in ISO and forskolin treated cardiomyocytes. Cells were loaded with Fura2-AM, paced at 1 Hz and treated with 100 nM ISO or 10 μM forskolin with subsequent applications of PDE inhibitors rolipram 10 μM, 8-MMX 30 μM and IBMX 100 μM. Shown are baseline Ca^2+^ amplitudes (black bars) and systolic Ca^2+^ transient amplitudes (grey bars) measured in paced **(A)** and resting **(B)** cells. Means ± SEM, *—p<0.05; **—p<0.01; ***—p<0.001 with ANOVA (compared to control, second value after / as compared to previous stimulation) followed by the Gasser-Greenhouse correction. n = 7 for ISO and n = 9 for forskolin cells in A, and n = 4 and 5 for ISO and forskolin cells in B, respectively (all isolated from at least 3 mice for each condition). Effects of ISO and forskolin in A are significantly different (p = 0.03 by one-way ANOVA).

To directly monitor how intracellular Ca^2+^ transients are modified under β-AR and forskolin stimulation during the experimental protocols mentioned above, we performed epifluorescence Ca^2+^ imaging using Fura2 under the same conditions. Both ISO and forskolin led to a strong increase of the calcium transient amplitude, while rolipram applied on top of ISO led to a further slight increase (Fig 5). Interestingly, forskolin increased Ca^2+^ transient amplitude much stronger than ISO, without any further subsequent effect of PDE inhibitors, suggesting than higher intracellular Ca^2+^ levels are generated after forskolin stimulation. This might potentially lead to a greater degree of PDE1 activation than after ISO treatment in paced cells.

## Discussion

**Fig 6:**
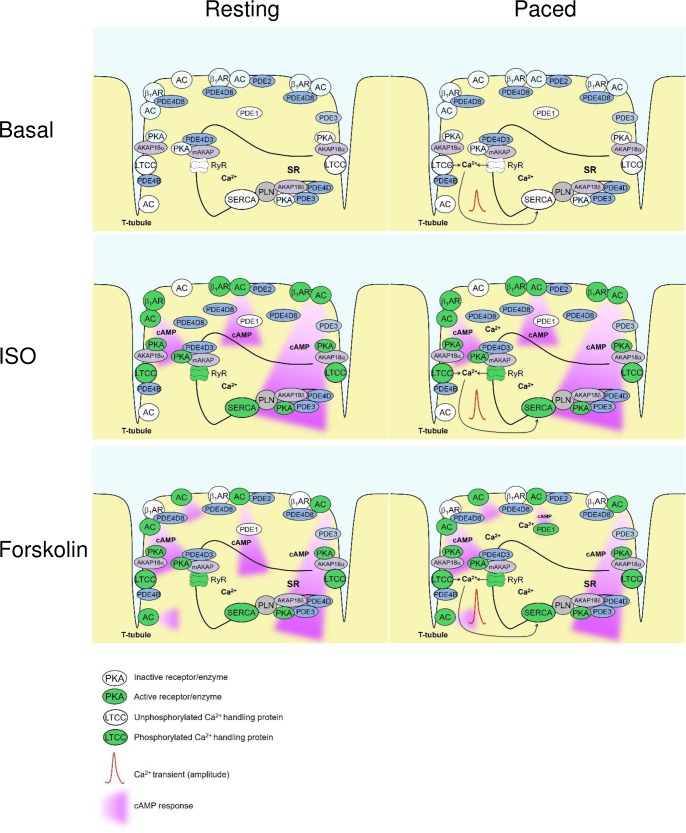
Schematic diagram highlighting Ca^2+^ and cAMP changes observed in this study under different experimental conditions (basal, ISO and forskolin stimulated cardiomyocytes with and without pacing). Pacing leads to an increase in Ca^2+^ levels which is further augmented by forskolin>ISO via PKA-dependent phosphorylation of Ca^2+^ handling proteins. However, pacing has little effect on cAMP levels, apart from the case when it is combined with forskolin stimulation, together both lead to PDE1 activation. Increase of PDE1 activity affects presumably a discrete subcellular microdomain which constitutes a small percentage of the whole cellular AMP content and can be therefore revealed in the cytosol only by the use of a PDE1 inhibitor. Forskolin and ISO generate quantitatively comparable but differently shaped amounts of cAMP which may come from ISO-induced dissociation of PDE4D8 from the β_1_-adrenergic receptor. This mechanism regulates local second messenger pool at the receptor and allows more rapid increase of cAMP in the cytosol, as compared to forskolin stimulation.

Previously, using classical biochemical assays and rapid freezing techniques, it has been shown that cAMP levels can rapidly change during each contraction cycle of the mouse heart [22]. However, it remained unclear to what extent these cAMP changes were associated with Ca^2+^ oscillations during cardiac ECC. Here we demonstrate that even if such rapid changes exist during contraction of isolated cardiomyocytes, they do not translate into sustained changes of cytosolic and submembrane AC and PDE activities under basal or cAMP-stimulating conditions in isolated mouse ventricular cardiomyocytes detected by FRET (see Figs 1, 3 and 4). A possible reason for this might be that the Ca^2+^ changes in field-stimulated cardiomyocytes are too small and too rapid to affect the overall AC activities which in turn, can translate into sustained changes in cytosolic or even local subcellular cAMP levels. Likewise, electrical pacing did not affect cardiomyocyte cAMP responses measured by FRET during stimulation with the β-AR agonist ISO and PDE4 inhibitors (see Figs 1 and 2). However, in sharp contrast, forskolin stimulation led to significantly more pronounced PDE1 inhibitor effects in paced myocytes (see Fig 3C and 3D), suggesting that the contractility-coupled Ca^2+^ pool can activate PDE1 under these, though supraphysiological but experimentally very important conditions. Interestingly, the kinetics of ISO responses were much faster than for forskolin (compare Fig 1C and 1E or Fig 3A and 3C). Together with relatively fast forskolin responses in the presence of IBMX (see Fig 2C) or after β_1_-AR stimulation [9], this suggests that ISO stimulation could potentially inhibit basal activity of some PDEs. Indeed, it has been previously shown that β_1_-AR forms a stable complex with PDE4D8 which dissociates upon ISO binding [23,24]. This mechanism was proposed as a way of local signal amplification and facilitation of the receptor-associated cAMP response [23], which could be detectable as a difference in signal kinetics in our system (see Fig 6).

Since there were no major differences in cAMP dynamics after β-AR stimulation in resting vs. paced cells, resting cardiomyocytes can be used for reliable analysis of real-time AMP dynamics by FRET or other comparable approaches under such stimulation protocols (see Figs 1, 2 and 4). In sharp contrast, when paced cardiomyocytes are treated with the direct AC activator forskolin, this leads to a significant increase of PDE1 associated cAMP hydrolysis, as revealed by the use of the PDE1 inhibitor 8-MMX. In this case, the effect of pacing could be mimicked by preincubation of resting cardiomyocytes with Ca^2+^ elevating agents such as thapsigargin or calcium ionophore A23187. These results are in line with the Ca^2+^ dependent stimulation of PDE1 hydrolytic activity. The absence of this effect after β-AR stimulation might be because of lower substrate (cAMP) levels or due to different compartmentation of forskolin- vs. β-AR-stimulated cAMP responses [25,26] which might differentially unmask elevated PDE1 activity due to increased Ca^2+^ flux [13]. However, our Ca^2+^ imaging data (Fig 5) strongly argue for the possibility that higher levels of PDE1 activity can be due to higher Ca^2+^ levels after forskolin stimulation, as compared to ISO stimulation. On the other hand, the absence of calcium ionophore and thapsigargin effects after ISO prestimulation (Fig 3B, right) argues for the fact that both strong calcium elevation and higher cAMP (substrate) levels after forskolin stimulation are important to boost PDE1 activity. Significantly more pronounced PDE1 inhibitor effects monitored under these conditions by FRET (see Fig 3C and 3D) indicate higher PDE1 activity in the intact cellular system during pacing. Interestingly, the overall amplitude of the forskolin response was not decreased under these conditions (Fig 3D). This might be because of compartmentation issues, i.e. PDE1 acting in one particular subcellular microdomain and forskolin increasing cAMP levels in a more global fashion. In addition, other more active and highly expressed PDE families such as PDE4 can potentially overwhelm and mask the effect of PDE1 which becomes detectable only upon inhibition of this latter PDE (see Fig 6). Although it could be helpful to directly measure PDE1 activity by classical biochemical assays (radioactive PDE activity assay, typically performed with heart lysates or large batches of isolated cardiomyocytes), this experiments lack spatial resolution and are difficult to perform due to low number of available cells after *in vitro* pacing protocol. In this case, FRET imaging represents a powerful approach for real-time cAMP monitoring in intact single cardiomyocytes.

## Conclusions

In summary, our results suggest that changes in intracellular Ca^2+^ during cardiomyocyte contraction can dynamically interact with cAMP levels under certain conditions. FRET-based imaging of cAMP dynamics in resting cells can be justified under β-adrenergic stimulation. However, under stronger global cAMP stimulation with forskolin and during the analysis of physiologically relevant effects of the Ca^2+^ activated PDE1, electric field stimulation is required to draw reliable conclusions.
